# Effects of two common polymorphisms in the 3' untranslated regions of estrogen receptor β on mRNA stability and translatability

**DOI:** 10.1186/1471-2156-10-55

**Published:** 2009-09-15

**Authors:** Milica Putnik, Chunyan Zhao, Jan-Åke Gustafsson, Karin Dahlman-Wright

**Affiliations:** 1 Department of Biosciences and Nutrition, Novum, Karolinska Institutet, S-141 57 Huddinge, Sweden

## Abstract

**Background:**

The present study represents the first attempt to functionally characterize two common single nucleotide polymorphisms (SNPs) in the 3'untranslated regions (3'UTRs) of estrogen receptor β (ERβ), focusing on the differences between alleles with regard to mRNA stability and translatability. These two ERβ SNPs have been investigated for association with disease in a large number of reports.

**Results:**

Here we examined allelic expression in breast tumor samples from heterozygous individuals. A significant difference in mRNA levels of the two alleles was observed for one of the SNPs. A cell model system was employed to further investigate potential molecular effects of the two SNPs. We used a modified plasmid, containing the ERβ promoter and ERβ 3'UTRs which include the different alleles of investigated SNPs. Quantitative Real-Time PCR was used to determine mRNA levels after inhibition of transcription by actinomycin D, and a luciferase assay was used to determine protein levels. The obtained results suggested that there was no difference in mRNA stability or translatability between the alleles of investigated SNPs.

**Conclusion:**

Our results indicate that observed associations between ERβ 3'UTR SNPs and disease susceptibility are due to linkage disequilibrium with another gene variant, rather than the variant itself being the susceptibility factor.

## Background

The steroid hormone estradiol-17β exerts its functions through binding to estrogen receptors (ERs), ERα and ERβ. The ERs belong to the nuclear receptor superfamily, a family of ligand-regulated transcription factors [1]. Both receptors, when ligand-activated, modulate gene expression and subsequently trigger a broad repertoire of physiological responses.

Estrogen signaling is involved in the regulation of development, growth and function of diverse systems, including human reproductive organs, mammary glands and skeletal and nervous systems. Aberrations in estrogen signaling have been proposed to be associated with several diseases, such as breast, endometrial, and ovarian cancers, osteoporosis, eating disorders and depression [2-4].

Five ERβ isoforms, designated ERβ1-5, have been reported in humans [5]. Among them, the presence of a corresponding protein has been clearly demonstrated only for ERβ1 (wild type) and ERβ2. The ERβ1-5 transcripts have unique sequences in place of exon 8, and thus different 3'untranslated region (3'UTRs). Single nucleotide polymorphisms (SNPs) in 3'UTRs have been identified only for ERβ1 and ERβ2. It has been shown that both ERβ1 and ERβ2 transcripts are regulated by the same promoter, designated as promoter 0N [6]. Promoter 0N was first described by Li et al. [7], and shown to contain both TATA box and initiator element (Inr) and putative binding sites for transcription factors AP-1, AML-1a and Oct-1.

**Figure 1:**
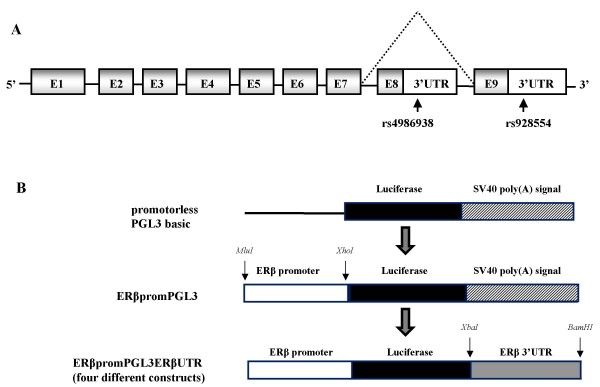
**Locations of the two SNPs in ERβ 3'UTRs and reporter constructs used in transient transfection assays**. **A**. rs4986938 is positioned in the 3'UTR of exon 8 and rs928554 in the 3'UTR exon 9. Exons are represented as shaded boxes, 3'UTRs as white boxes, and introns as connecting lines. Exon 9 is an alternatively spliced last exon (represented as a dashed line), which gives rise to the ERβ variant ERβ2. **B**. Luciferase reporter constructs used in transient transfection assays were generated from a promoterless PGL3 basic plasmid. The ERβ promoter was introduced using restriction enzymes *MluI *and *XhoI*. *XbaI *and *BamHI *were used to replace the SV40 poly(A) signal with ERβ 3'UTR sequences.

Two SNPs in the ERβ gene have been studied for association with a number of diseases. They are referred to as rs4986938 and rs928554 [8]. rs4986938 is a G↔A transition in exon 8, corresponding to ERβ1 3'UTR. rs928554 is a G↔A transition in exon 9, corresponding to ERβ2 3'UTR (Figure 1A). The distribution of these SNPs in some human populations is shown in Table 1 and Table 2.

**Table 1:** Frequency of rs4986938 in selected studies

**Studied populations**	**Sample size**	Allele frequency (%)*		**Study**
		**G**	**A**	
**Caucasians**	567	62.0	38.0	Westberg et al. [15]

**Caucasians**	1376	65.8	34.2	Kisiel et al. [16]

**Caucasians**	457	68.3	31.7	Forsti et al. [18]

**Caucasians**	628	65.0	35.0	Maguire et al. [19]

**Caucasians**	1107	62.0	38.0	Nilsson et al. [39]

**Caucasians**	1702	63.0	37.0	Ichikawa et al. [40]

**Caucasians**	862	62.0	38.0	Rexrode et al. [43]

**Asians**	526	86.5	13.5	Lee et al. [41]

**Asians**	776	86.0	14.0	Iwasaki et al. [44]

**South Americans**	758	67.0	33.0	Iwasaki et al. [44]

* Includes cases and controls

**Table 2:** Frequency of rs928554 in selected studies

**Studied populations**	**Sample size**	Allele frequency (%)*		**Study**
		**G**	**A**	
**Caucasians**	723	43.0	57.0	Maguire et al. [19]

**Caucasians**	1107	44.0	56.0	Nilsson et al. [39]

**Asians**	244	38.6	61.4	Maruyama et al. [17]

**Asians**	747	38.0	62.0	JSPN [45]

* Includes cases and controls

rs4986938 was first reported in a study on anorexia nervosa by Rosencrantz et al. [9]. rs928554 was first described by Nilsson et al., and both this SNP and rs4986938 were found to be associated with bulimia [10]. In another study, both SNPs have been associated with increased homocysteine levels [11]. Furthermore, rs4986938 has been associated with osteoporosis in women [12], ovulatory dysfunctions [13,14], Parkinson's [15] and Grave's disease [16]. In one study, rs928554 was associated with preeclampsia [17]. The allele frequencies of these SNPs in some case control studies are presented in Table 3. This table shows that different alleles are associated with the highest disease incidence for different diseases.

**Table 3:** Allele frequencies of rs4986938 and rs928554 in cases versus controls

**SNP**	**Allele**	**Disease**	**Cases (%)**	**Controls (%)**	**Study**
**rs4986938**	A	ovulatory dysfunctions	25.8	10.3	Sundarrajan C et al. [13]

**rs4986938**	G	Parkinson's disease	75.0	60.7	Westberg et al. [15]

**rs4986938**	A	Grave's disease	38.0	32.7	Kisiel et al. [16]

**rs4986938**	A	vascular dementia	51.0	38.0	Dresner-Pollak et al. [46]

**rs4986938**	G	bulimia	43.0	26.0	Nilsson et al. [10]
**rs928554**	G		61.0	43.0	

**rs928554**	A	preeclampsia	71.3	60.8	Maruyama et al. [17]

Studies of associations between these polymorphisms and breast cancer risk indicate that they do not have an effect *per se *[18-21]. However, association is found for haplotypes which contain either both SNPs [19,21] or rs4986938 solely [22].

3'UTRs are regulatory elements which can control protein expression, primarily through effects on mRNA stability but also through transcript translatability [23,24]. 3'UTRs control poly(A) tail length either through polyadenylation (due to the presence of polyadenylation AU signals) or deadenylation (e.g. binding of the PUF proteins) [25]. It has been reported that poly(A) tails correlate with translation, by poly(A) binding proteins interacting with translation factors at the 5' cap site. In most cases, long poly(A) tails are associated with induction and short poly(A) tails with repression of translation [24]. Loss of the poly(A) tail is thought to promote mRNA degradation by facilitating attack by both the exosome complex [26] and the decapping complex [27]. However to our knowledge, no studies have been published regarding mechanisms how SNPs regulate mRNA stability and/or translatability.

Recent studies show that 3'UTRs contain targets for micro RNAs (miRNAs) [28]. miRNAs are short endogenous RNAs (~ 23 nt) that play important gene-regulatory roles by pairing to the mRNAs of protein-coding genes to direct their posttranscriptional repression, suggesting an additional mechanism for 3'UTR-controlled protein expression.

In this study, we first examined whether ERβ mRNAs, corresponding to the different investigated alleles, were expressed at equal levels in breast tumor samples from heterozygous individuals. Both ERβ variants are expressed at significant amounts in breast tissue [29,30], which makes it a good system to study endogenous expression of ERβ mRNA. It is also generally acknowledged that ERβ has an important role in the development of this disease [31]. Next, we used an *in vitro *system to investigate if different alleles of the two commonly assayed SNPs in the ERβ 3'UTRs display differences in transcript stability or translatability, thus providing a molecular explanation for the observed disease associations.

## Methods

### Allelic expression assay

Primary breast tumor tissue samples from patients with invasive ductal carcinoma and undergoing breast cancer surgery were frozen in liquid nitrogen immediately after resection and stored at -80°C until use.

Genomic DNA from these samples was extracted using GenElute™ Mammalian Genomic DNA Miniprep Kit (SIGMA). RNA was extracted using Invitrogen's TRIzol LS Reagent protocol for mammalian tissues. 1 μg of total RNA was treated with Invitrogen's DNase I (Amplification Grade) to eliminate the remains of DNA, and then converted into cDNA using Invitrogen's protocol for SuperScript^® ^III Reverse Transcriptase.

To amplify the 3'UTRs that contain the studied SNPs, in both genomic DNA and cDNA, PCR was performed. The basic PCR protocol for Invitrogen's Taq DNA Polymerase was used, with specific primers: ERβ1-F (5'-TGCTGCTGGAGATGCTGAAT-3') and ERβ1-R (5'-TCACACCGACTCCTGAGAGTTG-3') for ERβ1 3'UTR, and ERβ2-F (5'-GGGCAGAAAAGGCCTCTCA-3') and ERβ2-R (5'-GAAGCCTCAGCTTTCTACATTGG-3') for ERβ2 3'UTR. The amplified fragments were sequenced on Applied Biosystems' 3730 × l DNA Analyzer in Macrogen DNA Sequencing Service [32].

Peak heights from sequencing reactions of genomic DNA and cDNA were measured for each allele for the two SNPs. Twenty samples were analyzed, and for each SNP under investigation five showed to be heterozygotes, i.e. informative for the assay. For each sample and SNP, the relative height of the peak for the cDNA versus the genomic DNA was determined for a particular allele.

### ERβ-Promoter-Luciferase-3'UTR-reporter constructs

Plasmid constructs were generated by cloning the ERβ promoter and different ERβ 3'UTR sequences into a promoterless PGL3 basic plasmid (Promega, Madison, W1).

A 1081 bp fragment of ERβ promoter 0N was amplified from human genomic DNA (Roche), using Expand Long Template PCR System (Roche, 150 U; recommended for fragments longer than 1 kb) and specific forward (5'-TATT**ACGCGT**TCCTGCTGGGGTGGGTGAG-3') and reverse primers (5'-TTAT**CTCGAG**CGAAGGGGCGCTTACCTT-3'). The primers were designed to incorporate restriction sites for enzymes MluI and XhoI (marked bold in the primer sequences), respectively. The promoterless PGL3 basic plasmid and the amplified fragment were digested with MluI and XhoI. The amplified fragment was inserted into the MluI/XhoI restriction sites of PGL3 basic, creating plasmid ERβpromPGL3 (Figure 1B).

A 933 bp fragment of ERβ exon 8, corresponding to ERβ1 3'UTR, was amplified from human genomic DNA (Roche), using Invitrogen's Platinum^® ^Taq DNA Polymerase protocol (5 U/μl), and forward (5'-TATT**TCTAGA**GCAGCCCGGCAGAGGACAG-3') and reverse primers (5'-TTAT**GGATCC**CCACATTGCCCCAGGGAAACACT-3'), containing restriction sites for enzymes XbaI and BamHI (marked bold in the primer sequences), respectively. The ERβpromPGL3 plasmid and the ERβ1 3'UTR fragment were digested with XbaI and BamHI. The ERβ1 3'UTR fragment was inserted into the XbaI/BamHI restriction sites of the ERβpromPGL3 plasmid, replacing the SV40 poly(A) signal, and creating plasmid ERβpromPGL3ERβ1UTR.

A 1380 bp fragment of ERβ exon 9, corresponding to ERβ2 3'UTR was amplified from human genomic DNA (Roche), using Roche's Expand Long Template PCR System (same protocol as above), and forward (5'-TATT**TCTAGA**GAAAAGGCCTCTCAAACACTC-3') and reverse primers (5'-TTAT**GGATCC**TAAATGCCAAACTACCGACTT-3'), containing restriction sites for enzymes XbaI and BamHI (marked bold in the primer sequences), respectively. The amplified fragment was inserted into the ERβpromPGL3 plasmid (same as above), creating plasmid ERβpromPGL3ERβ2UTR (Figure 1B).

Both amplified and cloned fragments contain the G nucleotide at the respective SNP positions. Mutagenesis was performed in order to generate the corresponding A alleles, using the QuikChange^® ^Site-Directed Mutagenesis Kit (Stratagene, La Jolla, California) and two primer sets: ERβ1MutF (5'-GCCCACAGAGGTCACAAGCTGAAGCGTGAACTC-3') and ERβ1MutR (5'-GAGTTCACGCTTCAGCTTGTGACCTCTGTGGGC-3') for ERβ1 3'UTR, and ERβ2MutF (5'-CAATGATCCCAGAGGGAAATTGAAGTGAAAATGTTACCC-3') and ERβ2MutR (5'-GGGTAACATTTTCACTTCAATTTCCCTCTGGGATCATTG-3') for ERβ2 3'UTR. The final products were four different constructs, two for the each studied SNP: ERβpromPGL3ERβ1UTR-G and ERβpromPGL3ERβ1UTR-A for rs4986938, and ERβpromPGL3ERβ2UTR-G and ERβpromPGL3ERβ2UTR-A for rs928554. The sequences of each pair of constructs differed only in the SNP under investigation. The sequences of all constructs were verified by DNA sequencing.

### Transient transfection assays

Luciferase activity was measured in three human cell lines - human fetal kidney HEK293 cells, cervical cancer HeLa cells and breast adenocarcinoma MCF7 cells. HEK293 and HeLa represent ER-negative cell lines [33,34], whereas MCF7 is an ER-positive cell line. The cells were seeded into 24-well plates (70% confluence) and co-transfected with the constructs incorporating different ERβ 3'UTRs (0.8 μg/well) and pRL-TK (Renilla plasmid, Promega, 0.02 μg/well), as internal control, using Lipofectamine 2000 (Invitrogen). Cells were harvested 24 h after transfection, and the activities of Firefly and Renilla luciferases were measured by the Dual Luciferase Reporter Assay System (Promega), using a Berthold FB12 Luminometer.

### mRNA stability assay

The assay was performed on the HEK293 cell line. The cells were maintained in 50% of Dulbecco's modified Eagle's medium (Gibco), supplemented with 10% fetal bovine serum (Saveen Werner AB), 1% penicillin-streptomycin (Gibco), and 50% F12 Nutrient Mixture with L-glutamine (Gibco), at 37°C in 5% CO_2_. Cells were transfected with a total amount of 10 μg DNA at 90-95% confluence in 10-cm dishes, using Lipofectamine 2000 (Invitrogen). At the end of transfection (4-6 h after Lipofectamine treatment), cells were split into 6-well plates. Twenty-four hours later, cells were treated with 1 μg/ml actinomycin D (Sigma) [35] to suppress transcription. Cells were harvested 0 and 24 h after actinomycin D treatment. Total RNA was extracted using the RNeasy kit (Invitrogen). mRNA levels were assayed using the SYBRGreen PCR master mix (Applied Biosystems), with specific forward (5'-ATCCGGAAGCGACCAACG-3') and reverse primers (5'-CGGTAAGACCTTTCGGTACTTC-3'), which target the Luciferase part of the Luciferase-ERβ3'UTR hybrid constructs. 18S rRNA levels, used for normalization, were measured using the Universal TaqMan PCR Master Mix and 20 × 18S Target Primers and Probe (all from Applied Biosystems). All Real-Time PCR experiments were performed on a 7500 Fast Real-Time PCR System (Applied Biosystems).

## Results

### mRNA levels of endogenous ERβ alleles differ in heterozygous carriers for rs4986938, but not in heterozygous carriers for rs928554

**Figure 2:**
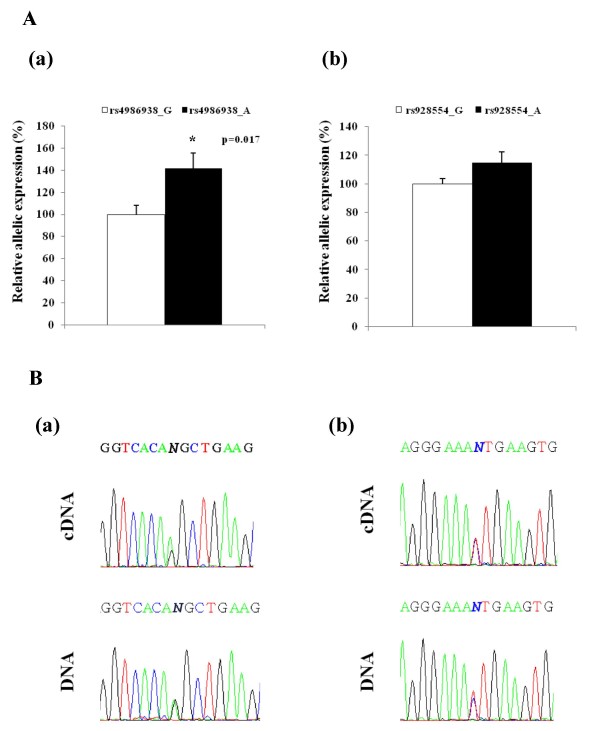
**Allelic expression of ERβ 3'UTR SNPs in breast tumor samples**. **A**. Genomic DNA was sequenced in two independent assays. cDNA synthesis was performed twice and each cDNA was sequenced in two independent assays. For each sample and allele, the average peak heights from the four cDNA sequencing assays were normalized by the average peak heights from the two DNA sequencing assays. The results from five breast tumor samples heterozygous for each SNP are presented as relative allelic ratios for cDNA versus genomic DNA. Data are shown as mean ± SD, with allele G set to 100%. **(a) **ERβ1 3'UTR polymorphism (rs4986938 G↔A); **(b) **ERβ2 polymorphism (rs928554 G↔A).**B**. Representative examples of genomic DNA and mRNA sequencing. **(a) **ERβ1 3'UTR polymorphism (rs4986938); **(b) **ERβ2 3'UTR polymorphism (rs928554). Observe that for rs928554 G↔A, the bottom strand was sequenced therefore the DNA sequence reads C and T, respectively.

To study a possible variation in allelic expression of endogenous transcripts in human tissues, we performed an allelic expression assay. In this assay, the relative mRNA levels of two alleles are determined by cDNA sequencing, and corrected for any difference in sequencing efficiency by normalizing with the sequence of genomic DNA from the same individuals [36]. A difference in relative mRNA levels for two alleles shows that the alleles differ with regard to mRNA levels, which could be the result of differences in transcription or mRNA stability. Figure 2 shows that there is a significant difference in allelic expression of rs4986938, but not of rs928554.

### ERβ 3'UTR alleles display similar luciferase activities and mRNA stability

To study molecular effects of the different alleles of the 3'UTRs, we generated four plasmids with different alleles at investigated SNP positions. The created plasmids contained the ERβ promoter, which regulates the expression of the reporter luciferase gene fused to 3'UTRs from ERβ1 or ERβ2, respectively (Figure 1B). A similar assay was successfully used in previous studies [35,37]. This assay would capture any differences in mRNA stability and translatability. The luciferase activities of all constructs were measured in three different cell lines.

**Figure 3:**
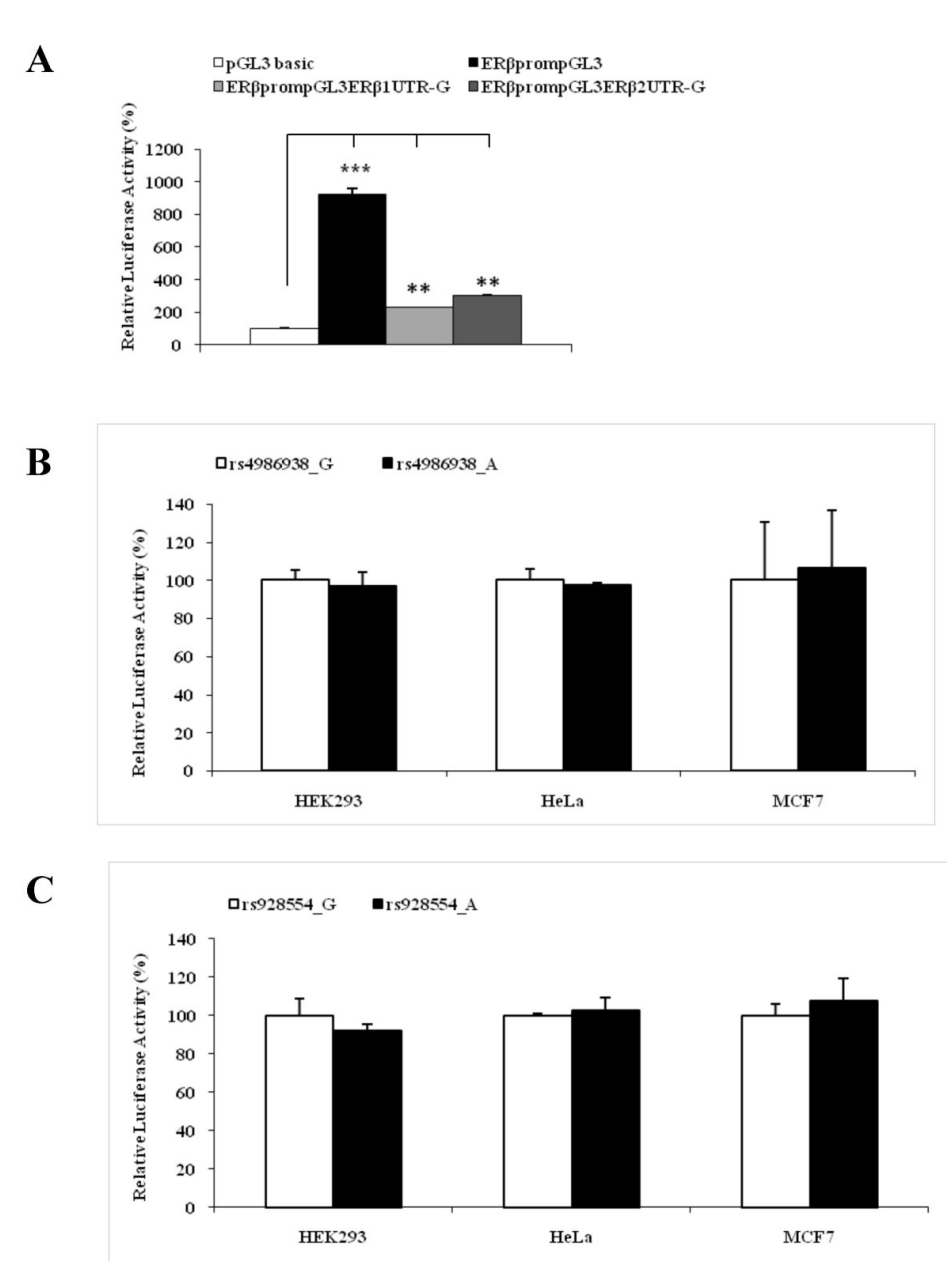
**Relative luciferase activities of 3'UTR constructs assayed in three different cell lines**. Relative luciferase activity is calculated as the ratio of Firefly luciferase activity vs. Renilla luciferase activity. Three human cell lines (HEK293, HeLa and MCF7) were co-transfected with 0.8 μg of the Firefly luciferase plasmids (PGL3 basic, ERβpromPGL3, ERβpromPGL3ERβ1UTRG/A for rs4986938, ERβpromPGL3ERβ2UTRG/A for rs928554) and 0.02 μg Renilla luciferase plasmid as internal control, at 70% confluence in 24-well plates. Cells were harvested 24 h after transfection, and the activities of Firefly and Renilla luciferases were measured. Each measurement represents the average of three independent assays using independent plasmid preparations. Data are shown as mean ± SD. **A**. The luciferase activity of generated constructs in HEK293 cells, is normalized to the basic PGL3 plasmid, whose activity is set to 100%. The same results were obtained in the two other cell lines (data not shown); **B**. ERβ1 3'UTR polymorphism (rs4986938 G↔A);**C**. ERβ2 polymorphism (rs928554 G↔A). The luciferase activity of allele G is set to 100%.

The luciferase activity of the plasmid ERβpromPGL3, which carries the ERβ promoter and SV40 poly(A) signal, was 9-fold higher comparing to the basic promoterless PGL3 vector, suggesting that the included ERβ promoter fragment is transcriptionally functional (Figure 3A). The luciferase activities of the constructs including the ERβ promoter and ERβ 3'UTR (ERβpromPGL3ERβUTR), were about 30% of that of ERβpromPGL3, i.e. 3-fold higher compared to the basic promoterless PGL3 vector. This is expected since the SV40 poly(A) signal, which has high translational potency, was replaced by the less potent 3'UTR.

There was no difference in luciferase activity between the two alleles of ERβ1 3'UTR, in any of the three analyzed cell lines, suggesting that the investigated ERβ1 3'UTR SNP does not influence the mRNA stability and translatability (Figure 3B). The same results were obtained for the two alleles of ERβ2 3'UTR (Figure 3C).

**Figure 4:**
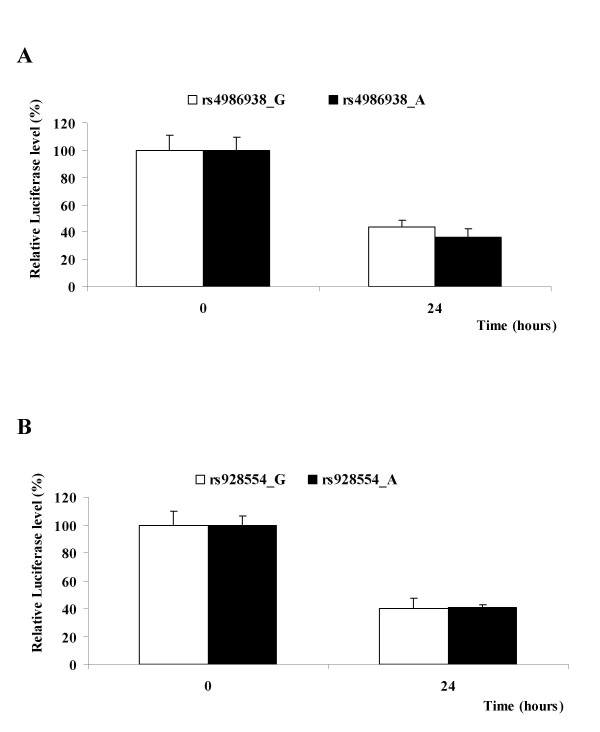
**ERβ 3'UTR mRNA stability in HEK293 cells**. The cells were transfected with 10 μg of the respective luciferase plasmids (ERβpromPGL3ERβ1UTRG/A for rs4986938, and ERβpromPGL3ERβ2UTRG/A for rs928554) at 90-95% confluence in 10-cm dishes. Twenty-four hours after transfection, cells were split into 6-well plates (6 wells/samples for each construct in total, corresponding to triplicate assays at two time points), so that each well could receive the same amount of plasmid. Twenty-four hours later, cells were treated with 1 μg/ml actinomycin D to suppress transcription. Cells were harvested 0 and 24 h after actinomycin D treatment, with triplicate samples for each time point. The whole experiment was performed twice, with reproducible results. One representative experiment is shown. Data are shown as mean ± SD, normalized to 18s, with the 0 h time point set to 100%. **A**. ERβ1 3'UTR polymorphism (rs4986938 G↔A); **B**. ERβ2 polymorphism (rs928554 G↔A).

In order to directly assay differences in mRNA stability between ERβ 3'UTR alleles, we determined mRNA levels in cells transfected with the different plasmids, after inhibition of transcription with actinomycin D. Luciferase constructs were transfected into HEK293 cells. Twenty-four hours later, cells were treated with actinomycin D to suppress transcription. mRNA levels at 0 and 24 h after actinomycin D treatment were quantified by Real-Time PCR. As shown in Figure 4, there was no difference in mRNA levels, after inhibition of transcription with actinomycin D, between the two alleles, for the two analyzed SNPs, suggesting that the investigated ERβ 3'UTR SNPs do not influence the mRNA stability.

### The predicted secondary structures of ERβ 3'UTR mRNAs suggest no differences in stability for the two alleles

We have investigated the secondary structures of ERβ 3'UTRs, using the Mfold web server for nucleic acid folding and hybridization prediction [38]. We compared ERβ 3'UTR sequences investigated in this study. Each of the four analyzed ERβ 3'UTRs can form a number of secondary structures. Focusing on the structures with the lowest free energy, i.e. the highest stability, no statistical difference was found between free energies for the G and A alleles of rs4986938, or for the G and A alleles of rs928554 (data not shown), supporting the results of the experimental mRNA stability assay.

## Discussion

SNPs that change the primary structure of the protein have not been reported for ERβ. However, there are reports of SNPs in promoter regions and 3'UTRs that could potentially alter mRNA and protein levels. In this study, we investigated the effects of two commonly studied SNPs, rs4986938 and rs928554, in the 3'UTRs of ERβ, on mRNA stability and translatability.

In order to study a possible variation in mRNA expression of transcripts that include the different alleles of the two SNPs, we have assayed mRNA levels in breast cancer samples which are heterozygotes for either of the two SNPs. Significant difference in mRNA expression of the different alleles was observed for the SNP in ERβ1 3'UTR, but not for the SNP in ERβ2 3'UTR.

ERβ SNPs have been found to have a large amount of linkage disequilibrium (LD) [21,39]. According to the HAPMAP haploblock structure, there are several additional SNPs surrounding rs498693, which are in LD with rs498693. Thus, we further investigated the molecular effects of this SNP independent from other polymorphisms using an *in vitro *assay, where investigated ERβ sequences are identical except for the SNP under investigation. Future studies should aim at investigating the effects of SNPs that are in LD with rs498693on mRNA levels.

The two ERβ SNPs were analyzed in cell lines that express or do not express endogenous ERs. We initially employed an assay that simultaneously detects differences in mRNA stability and translatability. This was complemented with an assay that directly detects differences in mRNA stability.

Luciferase reporter genes enable easy detection of changes in transcript and protein stability. Furthermore, studying isolated 3'UTRs in the context of a recombinant mRNA avoids confounding effects of additional genetic variations when assaying endogenous alleles. Also, measuring luciferase mRNA and protein as compared to ERβ mRNA and protein, avoids potential problems of distinguishing the molecules under investigation from any endogenous mRNA and protein. Neither of the two *in vitro *assays showed an effect of the different alleles of ERβ SNPs on mRNA stability or translatability.

It is also of importance to note that, although there are studies that associate these two SNPs with many different disorders, there are also studies which do not provide such evidence, even for the same disease. For example, our study of a Swedish cohort associated both rs4986938 and rs928554 with bulimia [10], while a study of a German cohort did not [9]. An investigation of a Danish cohort of postmenopausal women associated rs4986938 with bone mineral density [12], whereas an American study of a cohort of premenopausal women did not [40]. One study associated rs4986938 with the risk of endometriosis in a Korean population, but not in a Japanese population [41].

There are many reasons which could explain the inconsistency between different studies in showing association of, or lack of association of, SNPs in the ERβ 3'UTR with a particular disease. In relation to the studies in this paper, which suggest that the effects observed for the SNP in ERβ1 3'UTR on mRNA levels could be due to LD with another variant affecting mRNA levels, the haploblock structure and thus the LD vary between different populations. If a SNP that has not been the focus of most studies causes a phenotype, depending on the LD in a particular population, it might or might not be in LD with the investigated SNPs.

Finally, 3'UTRs of many protein-coding genes are found to be targets for miRNAs, short RNAs that regulate repression of translation. According to the miRNA database [42], ERβ can be a target for a number of miRNAs. However, none of them corresponds to the 3'UTR sequences where the SNPs in focus are located, although such miRNAs may exist, but remain to be identified.

## Conclusion

In summary, we have demonstrated a significant difference in allelic expression of rs4986938, but not of rs928554, in breast tumor tissues from heterozygous individuals. Our studies do not support the hypothesis that the two commonly studied ERβ SNPs change estrogen signaling by affecting ERβ mRNA stability or protein translatability. Combined, our results suggest that the observed associations between ERβ 3'UTR SNPs and disease susceptibility are due to LD with another gene variant effecting mRNA levels.

## Authors' contributions

MP carried out the experimental work and drafted the manuscript. CZ and KDW designed the study, supervised the experimental work and helped to draft the manuscript. JAG participated in the coordination of the study and drafting of the manuscript. All authors read, edited and approved the final manuscript.
